# An Exploratory Study of Serum Vasorin Levels in Polycystic Ovary Syndrome: A Novel Potential Biomarker for Diagnosis and Pathogenesis

**DOI:** 10.3390/metabo15030182

**Published:** 2025-03-09

**Authors:** Betül Keyif, Engin Yurtçu, Alper Başbuğ, Ali Yavuzcan, Fikret Gokhan Goynumer

**Affiliations:** 1 Department of Obstetrics and Gynecology, Faculty of Medicine, Duzce University, 81600 Duzce, Turkey; enginyurtcu@duzce.edu.tr (E.Y.); alperbasbug@duzce.edu.tr (A.B.); goynumergo@gmail.com (F.G.G.); 2 Department of Obstetrics and Gynecology, Faculty of Medicine, Sağlık Bilimleri University, Ankara Bilkent City Hospital, 06800 Ankara, Turkey; draliyavuzcan@yahoo.com

**Keywords:** biomarker, hormonal dysregulation, metabolic dysfunction, polycystic ovary syndrome, vasorin

## Abstract

**Objective**: This study aims to investigate the potential role of vasorin as a novel biomarker in the pathogenesis of polycystic ovary syndrome (PCOS) by evaluating serum vasorin levels in women diagnosed with PCOS. **Methods**: A prospective study was conducted at Düzce University Faculty of Medicine between March and July 2024, including 92 women with PCOS, diagnosed based on the 2003 Rotterdam criteria, and 68 age- and BMI-matched healthy controls. Serum vasorin levels were measured using an enzyme-linked immunosorbent assay (ELISA) and compared between the two groups. Additionally, correlations between vasorin levels and metabolic, inflammatory, and hormonal parameters were analyzed. **Results**: Women with PCOS had significantly lower serum vasorin levels (median: 0.70 pg/mL) compared to the control group (median: 2.36 pg/mL, *p* < 0.001). No significant correlation was found between vasorin and metabolic or hormonal parameters in the PCOS group. However, a weak positive correlation with prolactin was observed in the control group (r = 0.264, *p* = 0.030). Although vasorin is involved in inflammatory and oxidative-stress pathways, its association with insulin resistance and lipid metabolism remains unclear based on this study. Receiver Operating Characteristic (ROC) curve analysis demonstrated a high diagnostic performance for vasorin in distinguishing PCOS from healthy individuals (AUC = 0.918, *p* < 0.001, 95% CI: 0.869–0.967). The optimal cutoff value for vasorin (1.285 pg/mL) yielded 92.6% sensitivity and 87.0% specificity. **Conclusions**: These findings suggest that vasorin may serve as a promising biomarker for PCOS, potentially linking hormonal dysregulation, inflammatory responses, and ovarian dysfunction. However, further validation is required through longitudinal studies, multi-center cohorts, and mechanistic investigations. Additionally, comparative assessments with established biomarkers such as anti-Müllerian hormone (AMH) and androgen levels are warranted to determine vasorin’s diagnostic and prognostic utility in clinical practice.

## 1. Introduction

Polycystic ovary syndrome (PCOS) is one of the most prevalent endocrine disorders in women of reproductive age, affecting approximately 5% to 15% of women worldwide [1,2]. The syndrome is characterized by a wide spectrum of clinical manifestations, including ovulatory dysfunction, hyperandrogenism, and polycystic ovarian morphology detected via ultrasonography. Beyond its impact on reproductive health, PCOS is increasingly recognized as a multisystem disorder with metabolic, cardiovascular, and inflammatory consequences [3,4].

PCOS is a highly heterogeneous condition, making its diagnosis and classification complex. The 2003 Rotterdam criteria, widely used for PCOS diagnosis, require the presence of at least two out of three key features: ovulatory dysfunction (anovulation or oligomenorrhea), biochemical and/or clinical hyperandrogenism, and polycystic ovarian morphology on ultrasound [5,6]. However, due to the phenotypic variability of PCOS, no single biomarker has been universally accepted for its diagnosis. Clinically, anti-Müllerian hormone (AMH), androgen levels, and insulin resistance markers have been proposed as potential diagnostic tools, yet their utility remains inconsistent across different patient populations [7,8].

Emerging evidence suggests that chronic low-grade inflammation and oxidative stress play central roles in PCOS pathophysiology [9,10]. Identifying novel biomarkers that bridge the metabolic, hormonal, and inflammatory pathways is critical for enhancing early diagnosis, risk stratification, and targeted therapy in PCOS patients. In this context, vasorin, a transmembrane protein initially identified in a mouse kidney cDNA library in 2004, has gained attention for its involvement in TGF-β signaling, vascular remodeling, and oxidative stress modulation [11,12,13].

Vasorin is primarily expressed in vascular smooth muscle cells and has been found to regulate TGF-β signaling, a pathway implicated in fibrosis, inflammation, and oxidative stress [12,13]. It is also known to be expressed in granulosa cells and upregulated by luteinizing hormone (LH), suggesting a potential role in follicular survival and ovarian homeostasis [14,15]. Although its function in the human reproductive system remains largely unexplored, recent studies have reported vasorin expression in ovarian and endometrial tissues, raising the possibility of its involvement in PCOS pathogenesis [16,17]. However, serum vasorin levels in PCOS patients have not been previously investigated, and its relationship with metabolic, inflammatory, and endocrine abnormalities remains unclear.

Thus, the primary aim of this study was to evaluate serum vasorin levels in women with PCOS compared to healthy controls and to assess its potential as a novel biomarker for PCOS diagnosis. Additionally, we sought to explore the associations between vasorin and key metabolic, hormonal, and inflammatory parameters, providing new insights into its clinical relevance in PCOS pathophysiology. Given the heterogeneity of PCOS phenotypes, understanding whether vasorin levels correlate with specific clinical subgroups may aid in refining diagnostic algorithms and identifying potential therapeutic targets.

## 2. Materials and Methods

### 2.1. Study Design and Participants

This prospective observational study was conducted between November 2024 and February 2025 at the Department of Obstetrics and Gynecology, Düzce University Faculty of Medicine. A total of 92 women diagnosed with polycystic ovary syndrome (PCOS) based on the 2003 Rotterdam criteria were included in the study. The control group consisted of 68 healthy women who had no history of PCOS and were matched with the PCOS group in terms of age and body mass index (BMI). All participants provided written informed consent before enrollment, and the study was conducted in accordance with the ethical principles outlined in the Declaration of Helsinki. Ethical approval was granted by the local ethics committee (approval date: 21 October 2024; decision number: 2024/173).

### 2.2. Inclusion and Exclusion Criteria

Patients in the PCOS group met at least two of the three diagnostic criteria outlined in the 2003 Rotterdam guidelines: ovulatory dysfunction (anovulation or oligomenorrhea), biochemical and/or clinical hyperandrogenism, and polycystic ovarian morphology on ultrasound [5]. Exclusion criteria included congenital adrenal hyperplasia, Cushing’s syndrome, virilizing tumors, androgen-secreting tumors, glucose intolerance, diabetes mellitus, hepatic or renal dysfunction, and cardiovascular diseases such as hypertension and dyslipidemia. Additionally, patients with systemic inflammatory or autoimmune diseases, including Hashimoto’s thyroiditis, rheumatoid arthritis, and systemic lupus erythematosus, were excluded [18]. To avoid confounding factors, individuals using oral glucocorticoids, hormonal contraceptives, or antidiabetic medications were also not included in the study.

To minimize selection bias, participants were matched for age and BMI. PCOS diagnosis was confirmed based on the 2004 consensus criteria established by the European Society of Human Reproduction and Embryology and the American Society of Reproductive Medicine. These criteria define PCOS based on the presence of at least two of the following: polycystic ovarian morphology (≥12 follicles measuring 2–9 mm or ovarian volume >10 cm^3^), ovulatory dysfunction (anovulation or oligomenorrhea), and hyperandrogenism (clinical: Ferriman–Gallwey score >8; biochemical: total testosterone and/or dehydroepiandrosterone sulfate [DHEA-S] above the 95th percentile). Oligomenorrhea was defined as a menstrual cycle longer than 45 days, and amenorrhea as the absence of menstruation for more than three consecutive months [18]. To ensure consistency and reliability, all ultrasound assessments were performed by the same clinician using standardized imaging protocols.

### 2.3. Data Collection and Anthropometric Measurements

Demographic data, anthropometric measurements, and metabolic and hormonal parameters were systematically recorded for all participants. BMI was calculated as weight (kg) divided by height squared (m^2^). Waist circumference was measured at the midpoint between the lowest rib and the iliac crest, while hip circumference was measured at the widest part of the hips. The waist-to-hip ratio (WHR) was determined by dividing waist circumference by hip circumference. All anthropometric measurements were conducted by an experienced physician.

Hirsutism was assessed using the Ferriman–Gallwey scoring system, where a score below 8 indicated no hirsutism, a score between 8 and 15 was classified as mild hirsutism, a score between 16 and 25 as moderate hirsutism, and a score above 25 as severe hirsutism [19]. Additionally, reproductive history, including the number of pregnancies and live births, was recorded.

### 2.4. Metabolic and Hormonal Assessments

Venous blood samples were collected from all participants after at least eight hours of fasting, specifically on the second or third day of their menstrual cycle at 9:00 a.m. Serum samples were separated and analyzed for metabolic and hormonal parameters. The hormonal profile included follicle-stimulating hormone (FSH), luteinizing hormone (LH), estradiol (E2), total testosterone (TT), prolactin, and thyroid-stimulating hormone (TSH). Metabolic markers assessed were glycohemoglobin (HbA1c), fasting blood glucose, fasting insulin, total cholesterol, low-density lipoprotein (LDL), high-density lipoprotein (HDL), triglycerides, and the triglyceride-glucose (TyG) index. Additionally, C-reactive protein (CRP) was measured as an inflammatory marker, while the FSH/LH ratio and vasorin levels were also evaluated.

Insulin resistance was determined using the Homeostatic Model Assessment for Insulin Resistance (HOMA-IR). This index was calculated by multiplying the fasting glucose level (measured in millimoles per liter) by the fasting insulin level (measured in microunits per milliliter), then multiplying the result by 0.055, and finally dividing by 22.5. A HOMA-IR value greater than 2.5 was considered indicative of insulin resistance [20].

### 2.5. Analytical Process and Quality-Control Measures

Serum vasorin levels were quantified using a commercially available enzyme-linked immunosorbent assay (ELISA) kit (Biorbyt, CAT. NO: orb777407, Cambridge, UK) following the manufacturer’s protocol. The limit of detection (LoD) and limit of quantification (LoQ) for vasorin were 0.1 pg/mL and 0.3 pg/mL, respectively. To ensure the accuracy and reliability of the results, all assays were performed in duplicate, and the intra-assay and inter-assay coefficients of variation (CV%) were maintained below 10%.

The laboratory follows internal quality-control (CQI) procedures, ensuring that assay performance is monitored regularly. Furthermore, the laboratory participates in external quality assurance (VEQ) programs, which provide independent validation of analytical accuracy and reproducibility. These measures enhance the reliability and standardization of the vasorin assay results.

### 2.6. Vasorin Measurement Procedure

Vasorin levels were measured using a sandwich enzyme-linked immunosorbent assay (ELISA) following the manufacturer’s protocol (Kit: VASN [Vasorin], Human, Biorbyt, CAT. NO: orb777407) [21]. The microtiter plate was pre-coated with an anti-human vasorin antibody. Standards or serum samples were then added, followed by the sequential addition of a biotinylated anti-VASN antibody and streptavidin–horseradish peroxidase. After incubation, tetramethylbenzidine substrate was introduced, resulting in a color change. The reaction was terminated using sulfuric acid, and optical density (OD) was measured at 450 nm. Vasorin concentrations were determined by comparing OD values to a standard curve [21].

To ensure the accuracy and reproducibility of measurements, all assays were performed in duplicate. Intra-assay and inter-assay coefficients of variation (CV%) were maintained within acceptable limits. Hemolyzed samples were excluded from the study to prevent measurement errors.

### 2.7. Statistical Analysis

Data were coded and analyzed using SPSS (Version 22 for Windows, SPSS Inc., Chicago, IL, USA). The normality of continuous variables was assessed using the Kolmogorov–Smirnov test. Since the data did not follow a normal distribution, continuous variables were presented as median (min–max values), while categorical variables were expressed as frequencies and percentages. Categorical variables were compared using the Chi-square test, and when the expected frequency was less than five, Fisher’s exact test was applied. Continuous variables between groups were compared using the Mann–Whitney U test. The correlation between vasorin levels and selected parameters was analyzed using Spearman’s correlation test.

The diagnostic performance of vasorin in predicting PCOS was evaluated using Receiver Operating Characteristic (ROC) curve analysis. The Area Under the Curve (AUC) was calculated, and statistical significance was determined at *p* < 0.05. An optimal cutoff value was identified, and the diagnostic accuracy of vasorin was assessed through sensitivity and specificity calculations. In all statistical comparisons, a *p*-value < 0.05 was considered statistically significant.

A priori power analysis was conducted using G*Power software (Aydin et al., 2022) to determine the minimum required sample size for detecting a significant difference between groups [21]. Based on an effect size of 0.80, an alpha level of 0.05, and a power of 0.80, the analysis indicated that a minimum of 62 participants was required per group to achieve adequate statistical power. To ensure robust statistical power and account for potential dropouts, 92 PCOS patients were included.

## 3. Results

This study included 92 PCOS patients and 68 non-PCOS control patients. The demographic and clinical characteristics of the PCOS and control groups are summarized in Table 1. There was a significant difference in age between the PCOS group (median age 22.0, range 18–40) and the control group (median age 29.0, range 20–48) with a *p*-value of <0.001. BMI values showed no statistically significant difference between the PCOS (median value 23.7, range 17.3–48.6) and control groups (median value 22.8, range 17.1–33.2), with a *p*-value of 0.135.

Regarding BMI categories, the proportion of obese individuals (BMI 30–39.9) was higher in the PCOS group (22.8%) compared to the control group (14.7%). However, there was no statistically significant difference in BMI category distribution between the two groups (*p* = 0.441). Waist circumference, hip circumference, and waist/hip ratio did not show significant differences between the groups (*p* = 0.053, *p* = 0.156 and *p* = 0.071, respectively) (Table 1).

In terms of hirsutism, the presence was significantly higher in the PCOS group with 41.3% of individuals having mild hirsutism and 38.0% having moderate hirsutism, while no cases were observed in the control group (*p* < 0.001). The Ferriman–Gallwey score (FGS) was significantly higher in the PCOS group (median 12.5, range 0–24) compared to the control group (median 3, range 0–7), with a *p*-value of <0.001 (Table 1).

Reproductive characteristics such as gravidity and parity also showed significant differences between the groups (both *p* < 0.001). The median HOMA-IR values were significantly elevated in the PCOS group (2.45, range 0.09–15.23) compared to the control group (1.70, range 0.40–11.80), with a *p*-value of 0.001. Furthermore, insulin resistance was present in 46.7% of the PCOS group compared to 27.9% in the control group, which was statistically significant (*p* = 0.0016) (Table 1). These findings indicate significant metabolic and clinical differences between individuals with PCOS and controls, particularly in terms of insulin resistance, obesity, and hirsutism.

The analysis revealed significant differences in several biochemical parameters between the PCOS and control groups (Table 2). The FSH levels were significantly lower in the PCOS group (median 5.59, range 0.54–49.35) compared to the control group (median 6.78, range 3.12–18.63) (*p* < 0.001). The LH levels were significantly higher in the PCOS group (median 7.77, range 0.43–57.00) compared to the control group (median 6.15, range 2.76–16.13) (*p* = 0.013). Similarly, estradiol levels in the PCOS group (median 38.38, range 5–341) were significantly higher than those in the control group (median 48.99, range 5–292) (*p* = 0.037). Total testosterone levels were also elevated in the PCOS group (median 0.34, range 0.05–0.89) relative to the control group (median 0.25, range 0.03–0.65) (*p* < 0.001). Insulin levels were notably higher in the PCOS group (median 11.08, range 0.44–73.45) compared to the control group (median 7.72, range 1.72–51.33) (*p* = 0.001), as were triglyceride levels (107.66 ± 54.59 in the PCOS group vs. 77.55 ± 34.99 in the control group, *p* < 0.001). The TyG index, indicating insulin resistance, was also significantly elevated in the PCOS group (median 4.50, range 3.02–5.20) compared to the control group (median 4.36, range 3.98–5.0) (*p* = 0.003).

**Figure 1:**
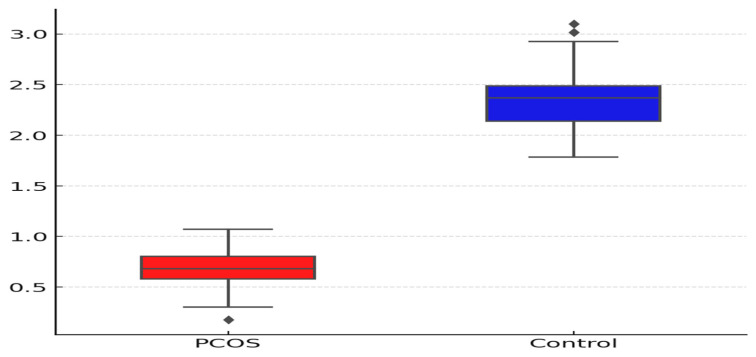
**Serum vasorin levels in groups.** The correlation between vasorin levels and various parameters, including age, BMI, FGS, HOMA-IR, TyG index, FSH, LH, E2, and other hormonal values, was evaluated. Correlation analysis revealed no significant linear relationship between vasorin and these parameters in the PCOS group (*p* > 0.05 for all comparisons). However, in the control group, a weak but statistically significant positive correlation was observed between vasorin and prolactin levels (correlation coefficient r = 0.264, *p* = 0.030).

The vasorin levels were significantly lower in the PCOS group compared to the control group (Table 2). Individuals in the PCOS group had a median vasorin level of 0.70 (range 0.35–4.36) pg/mL, whereas those in the control group had higher vasorin levels, with a median of 2.36 (range 0.90–4.78) pg/mL (Figure 1). This difference was statistically significant (*p* < 0.001), indicating that vasorin may have a distinct association with PCOS status.

**Figure 2:**
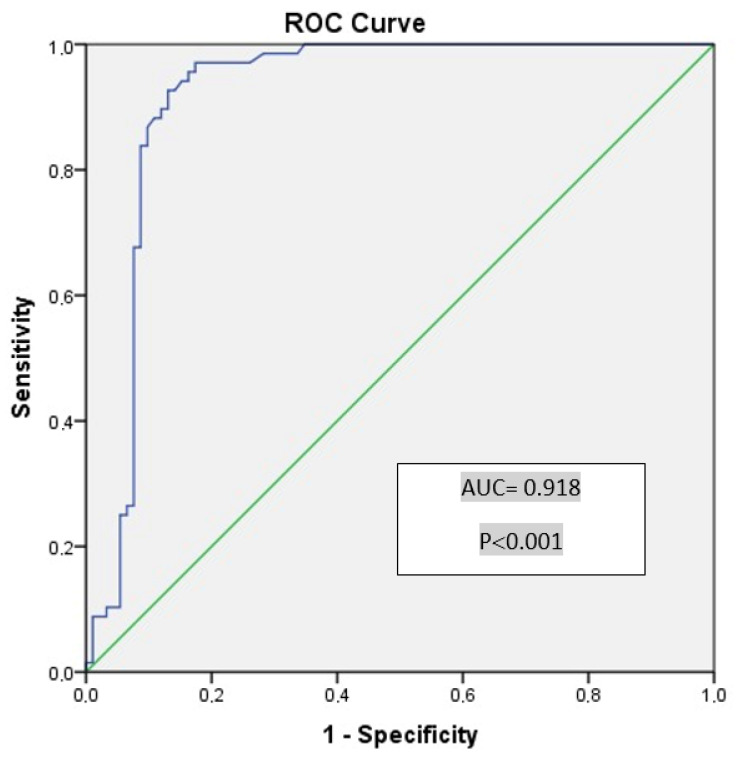
Receiver Operating Characteristic (ROC) curve of vasorin, delineating between healthy individuals and individuals with polycystic ovary syndrome.

The discriminative ability of vasorin levels in differentiating healthy individuals from those with PCOS was evaluated, and diagnostic results for specific cutoff values are presented in Table 3. The Area Under the Curve (AUC) for vasorin was calculated as 0.918 (Figure 2), indicating a statistically significant and excellent discriminative ability (*p* < 0.001, 95% CI: 0.869–0.967). When the cutoff value for vasorin was considered as 1.285, the sensitivity for detecting PCOS was 92.6%, while the specificity was 87.0% (Table 3).

## 4. Discussion

In this study, we investigated the potential role of vasorin as a novel biomarker in polycystic ovary syndrome (PCOS) and found that serum vasorin levels were significantly lower in PCOS patients compared to healthy controls. This finding suggests that vasorin may be involved in the pathophysiology of PCOS, particularly in relation to ovarian dysfunction, chronic inflammation, and hormonal imbalances [22]. Vasorin is known to modulate the Transforming Growth Factor-Beta (TGF-β) pathway, which plays a crucial role in ovarian folliculogenesis, tissue remodeling, and metabolic regulation. The observed decrease in vasorin levels in PCOS patients may indicate a disruption in TGF-β signaling, contributing to altered follicular development and inflammatory responses [23,24].

The role of TGF-β signaling in ovarian physiology is well established, particularly in regulating granulosa cell function, steroidogenesis, and follicular maturation. Dysregulation of this pathway has been implicated in PCOS, where excessive ovarian stromal fibrosis and anovulation are key pathological features [25]. Given vasorin’s function as a TGF-β inhibitor, reduced vasorin levels in PCOS may contribute to uncontrolled TGF-β activity, leading to excessive fibrotic changes and follicular arrest. This hypothesis aligns with previous studies showing increased ovarian fibrosis and altered extracellular matrix remodeling in PCOS patients. Future studies should explore whether restoring vasorin levels could counteract these fibrotic changes and improve ovarian function [26].

Additionally, chronic low-grade inflammation is recognized as a key component of PCOS pathogenesis [10]. Vasorin has been reported to exert anti-inflammatory effects by inhibiting oxidative stress-induced apoptosis and reducing reactive oxygen species generation. The observed reduction in vasorin levels in PCOS patients may indicate a disruption in this protective mechanism, potentially exacerbating inflammatory responses and contributing to metabolic dysfunction [27,28]. Although inflammatory markers such as TNF-α and IL-6 were not measured in this study, vasorin’s known anti-inflammatory properties suggest its potential role in modulating PCOS-related inflammation. Further research incorporating inflammatory markers is warranted to better understand this relationship [29].

Despite significant hormonal and metabolic abnormalities in the PCOS group, our study did not find a direct correlation between vasorin and insulin resistance parameters, including HOMA-IR and TyG index. This suggests that vasorin’s influence on PCOS may be more strongly associated with reproductive and inflammatory pathways rather than direct metabolic effects [30,31]. However, it is important to consider that our study was cross-sectional, and longitudinal investigations are necessary to determine whether vasorin levels fluctuate with disease progression, lifestyle modifications, or therapeutic interventions.

Interestingly, our study found a weak but statistically significant correlation between vasorin and prolactin levels in the control group, which was absent in the PCOS group. While prolactin has been linked to ovarian function and TGF-β modulation, its potential regulatory effect on vasorin remains unclear. Given that hyperprolactinemia is observed in some PCOS phenotypes, future studies should explore whether prolactin directly influences vasorin expression, potentially providing insights into PCOS heterogeneity.

One of the most striking findings of our study was the high diagnostic accuracy of vasorin in distinguishing PCOS patients from healthy individuals, with an AUC of 0.918. This suggests that vasorin may serve as a promising biomarker for PCOS diagnosis. However, its clinical utility should be carefully assessed in comparison with other established biomarkers, such as anti-Müllerian hormone (AMH), LH/FSH ratio, and androgen levels, which are currently utilized in PCOS diagnosis and classification [31,32].

Although our study demonstrates the potential of vasorin as a novel biomarker for PCOS, it is crucial to evaluate its specificity and reliability in different patient subgroups. For instance, vasorin levels may vary across different PCOS phenotypes, which were not separately analyzed in this study. Further investigations comparing vasorin levels between lean and obese PCOS patients, as well as among different PCOS subtypes, are necessary to determine whether it provides additional diagnostic value beyond existing biomarkers.

This study has several strengths, including its prospective design, well-matched control group, and rigorous statistical analyses. However, some limitations should be considered:

First, the cross-sectional nature of our study prevents us from establishing causality between vasorin levels and PCOS pathogenesis. Longitudinal studies are required to assess whether vasorin levels change over time or in response to treatment.

Second, although our findings suggest a potential role for vasorin in PCOS, we did not evaluate vasorin expression at the ovarian or endometrial tissue level. Future studies incorporating tissue-based analyses and mechanistic experiments will be critical in elucidating the exact role of vasorin in PCOS pathophysiology.

Third, our study population was limited to a single ethnic group, which may affect the generalizability of our findings. Larger, multicenter studies with diverse populations are needed to confirm our results.

Fourth, we did not perform a direct comparison between vasorin levels and androgen biomarkers such as total testosterone or dehydroepiandrosterone sulfate (DHEA-S). Given that hyperandrogenism is a hallmark of PCOS, future studies should explore the relationship between vasorin and androgen levels to assess its role in the hormonal dysregulation seen in PCOS.

Fifth, subgroup analyses based on PCOS phenotypes were not conducted in this study. Investigating whether vasorin expression differs across clinical variants of the disorder could provide further insight into its diagnostic and prognostic relevance.

Sixth, interleukin-6 levels were not measured in this study due to technical and logistical constraints. The available budget and laboratory resources were prioritized for vasorin analysis, which was the primary focus of our investigation. Additionally, due to sample volume limitations, simultaneous evaluation of multiple inflammatory markers was not feasible. Future studies should incorporate IL-6 and other inflammatory mediators to better elucidate the relationship between vasorin and inflammatory pathways in PCOS.

## 5. Conclusions

In conclusion, our study provides the first evidence that serum vasorin levels are significantly reduced in PCOS patients, highlighting its potential role as a biomarker for the disorder. While vasorin demonstrated high diagnostic accuracy, further validation in larger cohorts is necessary to establish its clinical applicability. Additionally, its relationship with established PCOS biomarkers, including androgens and AMH, should be explored in future studies.

Understanding the functional role of vasorin in PCOS may lead to the development of targeted therapies aimed at restoring its balance and mitigating the reproductive and metabolic disturbances associated with the syndrome. Future research should focus on exploring the interaction between vasorin, TGF-β signaling, and inflammatory pathways to provide deeper insights into the pathophysiology of PCOS.

## Figures and Tables

**Table 1:** An evaluation of the demographic characteristics of individuals in the PCOS and control groups.

Variables	PCOS (*n* = 92) Median (Lower-Upper)	Control (*n* = 68) Median (Lower-Upper)	*p* *
Age (year)	22.0 (18–40)	29.0 (20–48)	**<0.001 **
BMI (kg/m^2^)	23.7 (17.3–48.6)	22.8 (17.1–33.2)	0.135
BMI categories			
Underweight (<18.5)	3 (3.3%)	1 (1.5%)	0.441 **
Normal Weight (18.5–24.9)	44 (47.8%)	41 (60.3%)	
Overweight (25–29.9)	23 (25.0%)	16 (23.5%)	
Obese (30–39.9)	21 (22.8%)	10 (14.7%)	
Morbid Obese (>40)	1 (1.1%)	0 (0%)	
Waist circumference	84.5 (60.0–130.0)	77.5 (57.0–114.0)	0.053
Hip circumference	103.0 (80.0–138.0)	100.0 (86.0–127.0)	0.156
Waist/hip ratio	0.79 (0.66–0.98)	0.77 (0.65–0.94)	0.071
Waist/hip ratio risk			
Low (<0.80)	47 (51.1%)	41 (60.3%)	0.396 **
Medium (0.80–0.85)	21 (22.8%)	15 (22.1%)	
High (>0.85)	24 (26.1%)	12 (17.6%)	
FGS	12.5 (0–24)	3 (0–7)	**<0.001 **
**Hirsutizm** ** type **			
No hirsutism (<8)	19 (20.7%)	68 (100%)	<0.001 **
Mild hirsutism (8–15)	38 (41.3%)	0 (0%)	
Moderate hirsutism (16–25)	35 (38.0%)	0 (0%)	
Severe hirsutism (>25)	0 (0%)	0 (0%)	
Gravidity	0 (0–5)	0 (0–7)	**<0.001 **
Parity	0 (0–3)	0 (0–7)	**<0.001 **
HOMA-IR	2.45 (0.09–15.23)	1.70 (0.40–11.80)	**0.001 **
Insulin Resistance (Yes)	43 (46.7%)	19 (27.9%)	0.016 **
**PCOS Phenotype **			
Phenotype A (Ovulation Dysfunction + Hirsutism + Polycystic Ovary Morphology)	36 (39.1%)	-	
Phenotype B (Ovulation Dysfunction + Hirsutism)	11 (12.0%)	-	
Phenotype C (Hirsutism + Polycystic Ovary Morphology)	21 (22.8%)	-	
Phenotype D (Ovulation Dysfunction + Polycystic Ovary Morphology)	24 (26.1%)	-	

FGS: Ferriman–Gallwey score; BMI: body mass Index; HOMA-IR: Homeostatic Model Assessment for Insulin Resistance. *: Mann–Whitney U test; **: Chi square test.

**Table 2:** An evaluation of the biochemical parameters of individuals in the PCOS and control groups.

Variables	PCOS (*n* = 92) Median (Lower–Upper)	Control (*n* = 68) Median (Lower–Upper)	*p* *
FSH	5.59 (0.54–49.35)	6.78 (3.12–18.63)	**<0.001 **
LH	7.77 (0.43–57.00)	6.15 (2.76–16.13)	**0.013 **
E2	38.38 (5–341)	48.99 (5–292)	**0.037 **
Total Testosterone	0.34 (0.05–0.89)	0.25 (0.03–0.65)	**<0.001 **
Prolactin	19.19 (0.65–68.38)	16.94 (8.22–42.76)	0.106
TSH	1.87 (0.32–6.90)	1.91 (0.02–7.02)	0.912
HBA1C	5.05 (4.50–6.00)	5.10 (4.5–8.00)	0.499
Insulin	11.08 (0.44–73.45)	7.72 (1.72-51.33)	**<0.001 **
Fasting Glucose	90.60 (69.80–123.0)	91.15 (56.80–129.40)	0.556
Total Cholesterol	161.15 (0.25–284.40)	160.25 (105.00–237.0)	0.875
LDL	85.05 (7.46–163.00)	89.18 (39.38–151.06)	0.540
HDL	51.45 (26.90–97.80)	53.60 (28.60–90.40)	0.228
Triglyceride	91.40 (39.70–329.20)	70.65 (30.50–224.60)	**0.001 **
TyG Index	4.50 (3.02–5.20)	4.36 (3.98–5.0)	**0.003 **
CRP	0.16 (0.06–3.38)	0.14 (0.06–4.16)	0.176
FSH/LH	1.38 (0.19–5.70)	0.88 (0.34–3.40)	**<0.001 **
Vasorin pg/mL	0.70 (0.35–4.36)	2.36 (0.90–4.78)	**<0.001 **
Vasorin values for PCOS Phenotype			
Phenotype A	0.68 (0.35–1.80)		
Phenotype B	0.62 (0.40–2.13)		
Phenotype C	0.86 (0.39–2.64)		
Phenotype D	0.73 (0.41–4.01)		

* Mann–Whitney U test.

**Table 3:** The results of the Receiver Operating Characteristic (ROC) analysis evaluating the discriminative ability of vasorin levels between healthy individuals and those with PCOS.

Cutoff Value for Vasorin	Sensitivity (%)	Specificity (%)	AUC	SE	*p*	%95 CI (Min–Max)
0.98	97.1	73.9	0.918	0.025	**<0.001 **	0.869–0.967
1.09	97.1	82.6				
1.225	94.1	84.8				
1.285	92.6	87.0				
1.365	86.7	87.0				

AUC: Area Under the Curve; SE: Standard Error; CI: Confidence interval.

## Data Availability

The raw data supporting the conclusions of this article will be made available by the authors on request.

## Abbreviations

PCOS	Polycystic Ovary Syndrome
BMI	Body Mass Index
AMH	Anti-Müllerian Hormone
FSH	Follicle-Stimulating Hormone
LH	Luteinizing Hormone
E2	Estradiol
HOMA-IR	Homeostatic Model Assessment for Insulin Resistance
FGS	Ferriman–Gallwey Score
